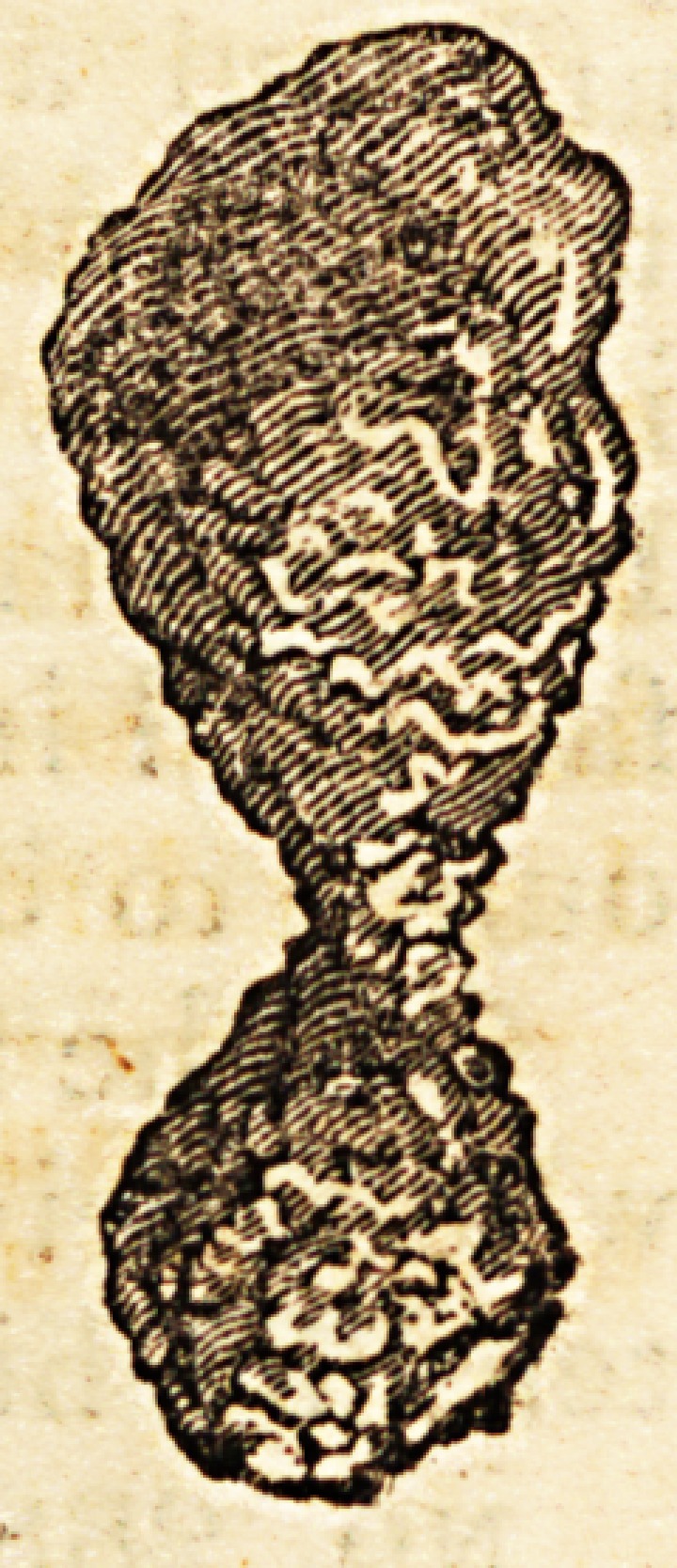# Case of Lithotomy, with Singularly-Shaped Calculus

**Published:** 1823-02

**Authors:** W. Money

**Affiliations:** Surgeon to the Asylum for the Recovery of Health, and to the Infirmary for Sick Children.


Art. VI.—*Case of Lithotomy, with singularly-shaped Calculus.* By W. Money, Esq. Surgeon to the Asylum for the Recovery of Health, and to the Infirmary for Sick Children.

James Snell, eleven years of age, came under my care at the Westminster station of the Metropolitan Infirmary for Children, in the early part of March, 1821. For several years, and even (according to his mother's belief) from his birth, he had suffered severely in his efforts to void his urine. His symptoms were strongly indicative of a calculus in the bladder: I therefore introduced the sound; but, though I made the examination with all the care and assiduity I could exert, I could not discover the presence of such a cause of his complaints. The symptoms were, however, too characteristic for me to relinquish my suspicion of the existence of a calculus; and I accordingly examined a second time, when I ascertained the presence of a stone. In subsequent examinations, unless I directed the sound to a particular point of the bladder, no calculus could be found. With the impressions arising from these investigations, I proceeded, on the 16th of March, to perform the lateral operation. On the introduction of my finger into the bladder, I found the calculus adhering to the parietes of that viscus on the left side, and near to its fundus,—or, as I should more properly say, lodged in a pouch at that part. I also readily felt that the figure of the calculus was irregular, and, on endeavouring to dislodge it with my finger, it broke; one, or the smaller portion, falling into the cavity of the bladder, whilst the other was retained in its pouch. In a very short time both portions were, however, extracted. The bladder was universally in a rugous state. Nothing else worthy of remark occurred in the case.

My young friend, Mr. W. Clift, at the College of Surgeons, has been so kind as to favour me with a drawing of the calculus; a cut of which is annexed.

My intentions in communicating this account to the public are to record the peculiar figure of the calculus; and to offer to surgeons an example of the importance of attention to symptomatology, as well as what relates to the manual practice of our art; since it was this which enabled me to discover the calculus on a second and very painful examination, after having failed in a former one, and when other surgeons had stated it to be their opinion that no stone existed, because they also had not been able to ascertain its presence.

---

ART. VII.—*A Case of Poisoning by Arsenic.* By J. W. EDWARDS, Esq. Surgeon.

## Figures and Tables

**Figure f1:**